# Soil column-experimental research on the migration pattern of petroleum pollutant in the soil

**DOI:** 10.1038/s41598-024-60588-4

**Published:** 2024-05-01

**Authors:** Bo Zhang, Meiyi Feng, Chao Du, Yuanhao Guo

**Affiliations:** 1 School of Mining Engineering, Heilongjiang University of Science and Technology, Harbin, 150022 China; 2 Heilongjiang Province Geophysical Survey Survey Institute, Harbin, 150036 China; 3 Heilongjiang Province Ecological Geology Survey and Research Institute, Harbin, 150036 China

**Keywords:** Petroleum pollutant, Migration pattern, Soil column, Undisturbed soil, Aromatic hydrocarbons, Daqing oilfield, Hydrology, Solid Earth sciences

## Abstract

In the process of oilfield exploitation and production, harmful pollutants, such as Crude oil that falls to the ground (generally refers to crude oil that leaks to the ground during oil production or transportation), production wastewater and oil-bearing mud are produced. In this contribution, the soil and crude oil from Daqing area are adopted as experimental materials to make a soil column-experimental device. The results show that the maximum migration depth of petroleum pollutants is 25 cm, most of the pollutants exist above 10 cm. The components of pollutants in disturbed soil column are complex, and the peak area of each component is large, mainly distributed in C_12_–C_28_, while in undisturbed soil column, the content of pollutants is small, and the peak area of each component is also small, mainly distributed in C_12_–C_22_. With the increase of depth, the relative content of aromatic hydrocarbons increases. The migration ability of low carbon component is weaker than the other components in crude oil. The components with high carbon number are significantly higher in shallow part. The relative contents of each component from high to low are saturates, aromatic hydrocarbons, resin and asphaltene in the soil. Compared with disturbed soil columns, the structure of undisturbed soil is complex, and the migration rate of pollutants in undisturbed soil is slower than that in disturbed soil. With the increase of depth, the light components of disturbed soil columns gradually decrease, and the relative content of heavy components changes little. The light components of the undisturbed soil column also gradually decreased, and the heavy components greater than C22 did not migrate to the depth of the soil column.

## Introduction

In the processes of exploitation and transportation, crude oil will enter and contaminate inevitably, and it changes structure and physicochemical properties of the soil thus leading to soil salinization^1^. Moreover, part of harmful component of crude oil enters the groundwater system further, resulting far-reaching effects. The presence of benzene and polycyclic aromatic hydrocarbons in crude oil poses potential risks of carcinogenicity, teratogenicity, and mutagenicity, which are of great harm to human health and the environment^2^. According to the statistical data, about 8 million tons crude oil are leaking every year, which makes severe impacts on the environment^3^. The pollution caused by crude oil has attracted more and more attention^4,5^.

It is of great significance to study the migration rule of petroleum pollutants in the soil, could provide a theoretical foundation to the investigation and restoration of soil pollution, so it has great scientific and practical significance^4,5^. According to in-situ chromatographic analyses, Li et al.^6^ proposed that the total concentration of petroleum hydrocarbons and the migration depth could be characterized by a negative exponential equation. The study conducted by Wang et al.^7^ involved a soil column experiment aimed at investigating the influence of injection quantity on oil release in the vadose zone. Zheng et al.^8^ designed a soil column simulation experiment for diesel oil, which showed that 85% of diesel oil remained in the depth of 0–10 cm, and 90% of crude oil remained in the soil layer above 20 cm. Zhang et al.^9^ proved that the crude oil of different components yield different migration patterns in the disturbed soil by soil column-experimental experiment. However, in these experiments, disturbed soil was used for experimental simulations, and the porosity and structure of soil were different from that of natural soil. In other words, the oil migration pattern in undisturbed soil may be different from that of disturbed soil which should be proven.

The Daqing oilfield has been exploited for decades, producing abundant pollutants such as waste oil in soil and oily sewage that pollute soil and groundwater. In this contribution, undisturbed soil column-experimental experiments compared to disturbed soil column-experimental experiments were carried out on soil from the Daqing oilfield. Based on the disturbed soil and undisturbed soil experiments, the effects of pollutant concentration and pouring amount on the migration pattern of petroleum pollutants in soil were studied. Compared with disturbed soil column, the structure of undisturbed soil is complex. Therefore, experiments were carried out on disturbed soil columns and undisturbed soil columns respectively, and the migration rule of pollutants in them is compared, in order to make the experimental results are closer to the migration rule of actual soil pollutants. In this paper, the migration pattern of petroleum pollutants in soil and their affecting factors were systematically studied.

## Study area and samples collection

### Study area

Daqing Oilfield is located in Songnen plain in the west of Heilongjiang province, Northeast China, with an altitude is 127–165 m. The petroleum reservoir rocks are Cretaceous continental sandstones, and the oil layer occurs in the large anticline structure, the occurrence depth is 900–1200 m. The study area belongs to the continental monsoon climate zone, which is influenced by the cold air from the Mongolia interior and the monsoon from warm ocean current. There is a rainy summer and dry winter in a year, with an average annual precipitation of 434 mm.

**Figure 1:**
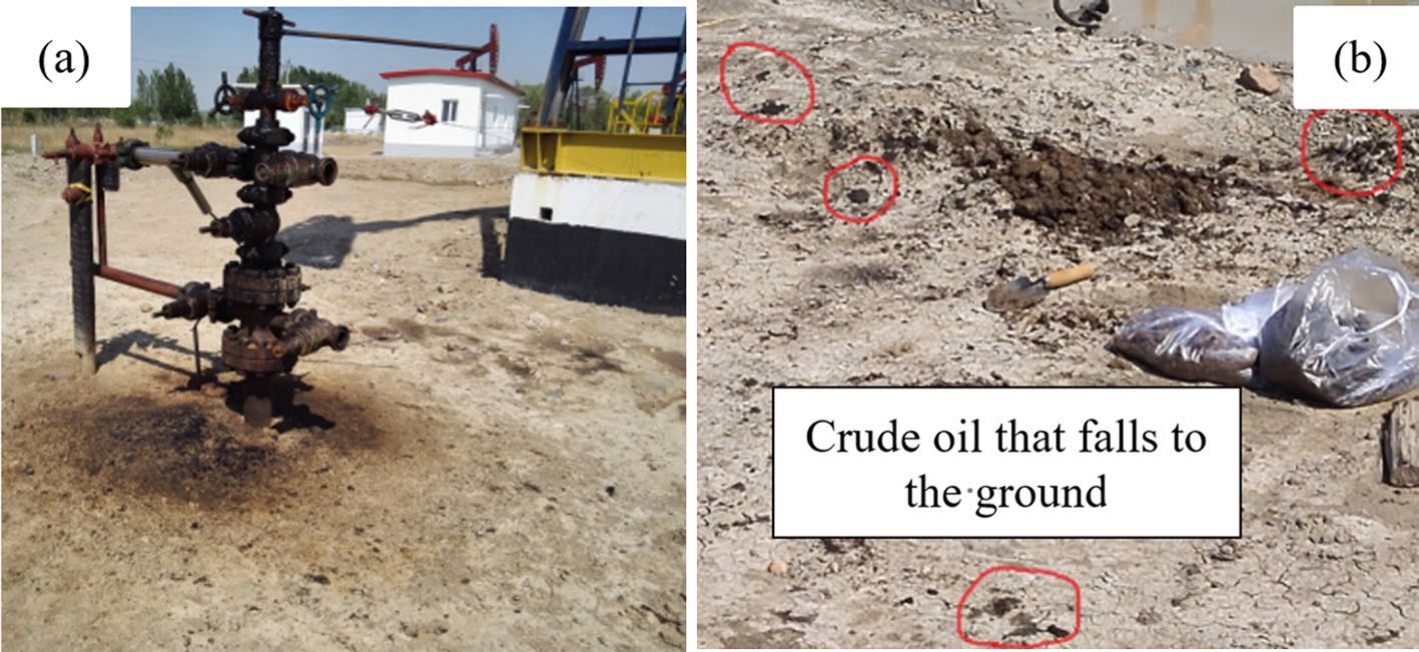
Crude oil contaminated soil. (**a**) Contaminated soil in wellhead; (**b**) Crude oil that falls to the ground.

Chernozem is the main soil type in the study area. Petroleum-contaminated soils are widely distributed in the oilfield region. Oil spots can be seen on the soil surface near the pithead (Fig. 1a and b). Some Crude oil that falls to the ground is often buried on site, which causes pollution to the environment. Pollutants are produced during drilling, oil production, underground operation, exploitation, and transportation.

### Samples collection

During sample collection, sections were scooped out to collect uncontaminated soil samples first. The soil was collected in parallel to the section, and 1.5 kg soil samples were taken every 5 cm depth for each section. And soil samples from 18 sections with the same depth were mixed evenly to make disturbed soil samples. For undisturbed soil samples, a 90 cm long soil sampler was adopted to cut samples. All soil samples were sealed to avoid exposure to air after collection.

Before the experiment, physical features of the soil sample, e.g. specific gravity, density, porosity, soil leached water pH value, water content, and organic matter content, were analyzed and the determination of soil leached water pH value selected the potentiometric method, specifically with water as the leaching agent, the ratio of water to soil is 2.5:1, and then the pH value of the soil leached water was measured by the potentiometric method. Results of soil features are shown in Table 1.

**Table 1:** Physical features of the soil sample.

Depth (cm)	Specific gravity	Density (g/cm^3^)	Porosity (%)	pH value of leached water	Water content (%)	Organic matter content (mg/g)
10	2.66	1.143	57.03	8.2–8.3	11.41	28.42
20	2.50	1.270	49.20		15.52	9.32
30	2.66	1.478	44.44		16.68	3.62
40	2.43	1.496	38.44		15.93	3.21

### Features of the crude oil

The crude oil from the Daqing oil field was used as the contaminant. The crude oil is composed mainly of complex mixtures of hydrocarbon components, which are black or dark brown oil at room temperature. The crude oil is composed of straight-chain alkanes with the molecular formula C_n_H_2n+2_, containing a small amount of unsaturated hydrocarbons. The density and kinematic viscosity of the oil is about 0.86 g/mL, and 22.2 mm^2^/s. The petroleum is composed of carbon (85.74%), hydrogen (13.31%), nitrogen (0.15%), sulfur (0.11%), and oxygen (0.69%). It has high wax content (20–30%), high freezing point, high viscosity and low sulfur content. Four-component analyses of petroleum show that saturates, aromatics, resin, and asphaltene of crude oil were 54.7, 27.8, 15.1 and 2.4%, respectively^10^. The above data are obtained by analyzing the physical and chemical properties of crude oil.

## Experimental methods

Based on the ultrasonic extraction technology and gas chromatography analysis technology, this study simulates the migration pattern of crude oil in soil during the precipitation process.

### Simulated rainfall-infiltration soil column experimental device

**Figure 2:**
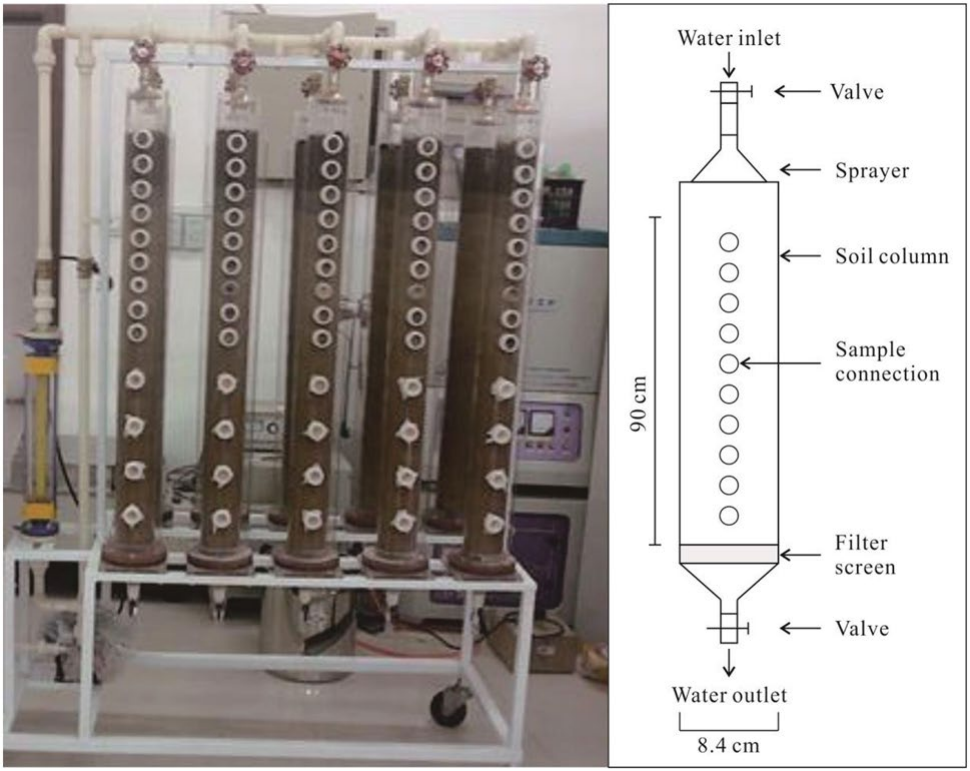
Soil column leaching device.

As shown in the Fig. 2, the soil column leaching device is composed of two parts: soil columns and water supply device. The soil columns are made of transparent cylindrical plexiglas tubes of 100 cm height, 10 cm outer diameter and 8.4 cm inner diameter. A stainless-steel filter screen is set at the bottom of the tube. The scale of each soil column ranges from 0 to 90 cm. There is a sample connection at the tube every 5 cm above the depth of 40 cm and every 10 cm below the depth of 40 cm. When filling the soil column, a layer of quartz sand was put at the bottom of the soil column to prevent soil loss. Ten columns were filled with disturbed soil, and four columns were filled with undisturbed soil. The water supply device is composed of a water tank (Ordinary laboratory stainless steel water tank), pump (SHB-III), flow meter (ACU20FD-L) and sprinkler head (JTG). In the experiment, 5 g, 10 g and 15 g of crude oil were added to different experimental soil columns. The simulating rainwater of pH value of 5.6 was prepared by adding a certain amount of hydrochloric acid in distilled water. The precipitation of 8 months (1600 mL), 24 months (4800 mL) and 40 months (8000 mL) were experimental. The flow rate was 4 × 10^−6^ to 360 × 10^−^6 m^3^/h, and the diameter of water droplets was 6–7 mm. At the same time, two blank soil columns without oil were set for comparison, using a precipitation amount representative of a duration of 8 months (1600 mL). The pouring was carried out 4 times with an interval of 5 days equally. After that, soil samples were then taken for composition analyses. Experimental parameters are listed in the Table 2.

**Table 2:** Experimental parameters.

NO.	1	2	3	4	5	6	7	8	9	Blan k 1	O1	O2	O3	Blan k 2
Oil mass (g)	5	5	5	10	10	10	15	15	15	0	5	10	15	0
Pouring amount (mL)	1600	4800	8000	1600	4800	8000	1600	4800	8000	1600	1600	1600	1600	1600

### Petroleum pollutant extraction methods

The ultrasonic extraction method is widely used to extract petroleum pollutant from soil^11,12^. During extraction, 3 g dried and grinded soil sample was prepared firstly. Then 30 mL compounded solvent of 4:1 acetone and dichloromethane were added. The ultrasonic vibration was conducted for 20 min at a temperature of 40 °C, using an ultrasonic power of 100 W. Then the sample was transferred into a centrifuge tube and centrifuged for 6 min at 4500 r/min to separate solids and liquids. The liquid should be collected and the extraction process repeated three times to ensure complete extraction. After completely evaporating the solvent, the oil pollutants can be obtained. The extraction rate of crude oil pollutant could reach 85.73% by the ultrasonic extraction method.

### Determination of petroleum pollutant components

The components of petroleum pollutant were analysed by gas chromatography and four-component analyses. Gas chromatography with hydrogen flame ionization detector (FID) was used, and the specific experimental method is described in Saari et al. and Gu et al.^13,14^.

For four-component analyses, 0.5 grams of extracted pollutant was added to 20 mL of petroleum ether. The insoluble part in petroleum ether solvent is asphaltene. The silica gel and alumina should be introduced into the adsorption column from the upper end, followed by gently tapping the column to ensure their even distribution. The adsorption column was immediately wetted by adding 30 mL of petroleum ether. The dissolved sample was then added, followed by pouring 80 mL of petroleum ether into the column as a rinse agent and collecting the rinse agent. The rinse agents were changed to a mixture of dichloromethane-petroleum ether in a ratio of 2:1, as well as anhydrous ethanol-chloroform, and both were collected. Subsequently, the collected rinse agents were placed in a constant temperature drying oven at 105 °C for 2 h. Finally, saturates, aromatics, resin, and asphaltene pollutants were obtained and weighed to calculate their respective percentages.

### Consent to participate

All the authors consent to participate.

## Results

### Results of disturbed soil column experiment

**Figure 3:**
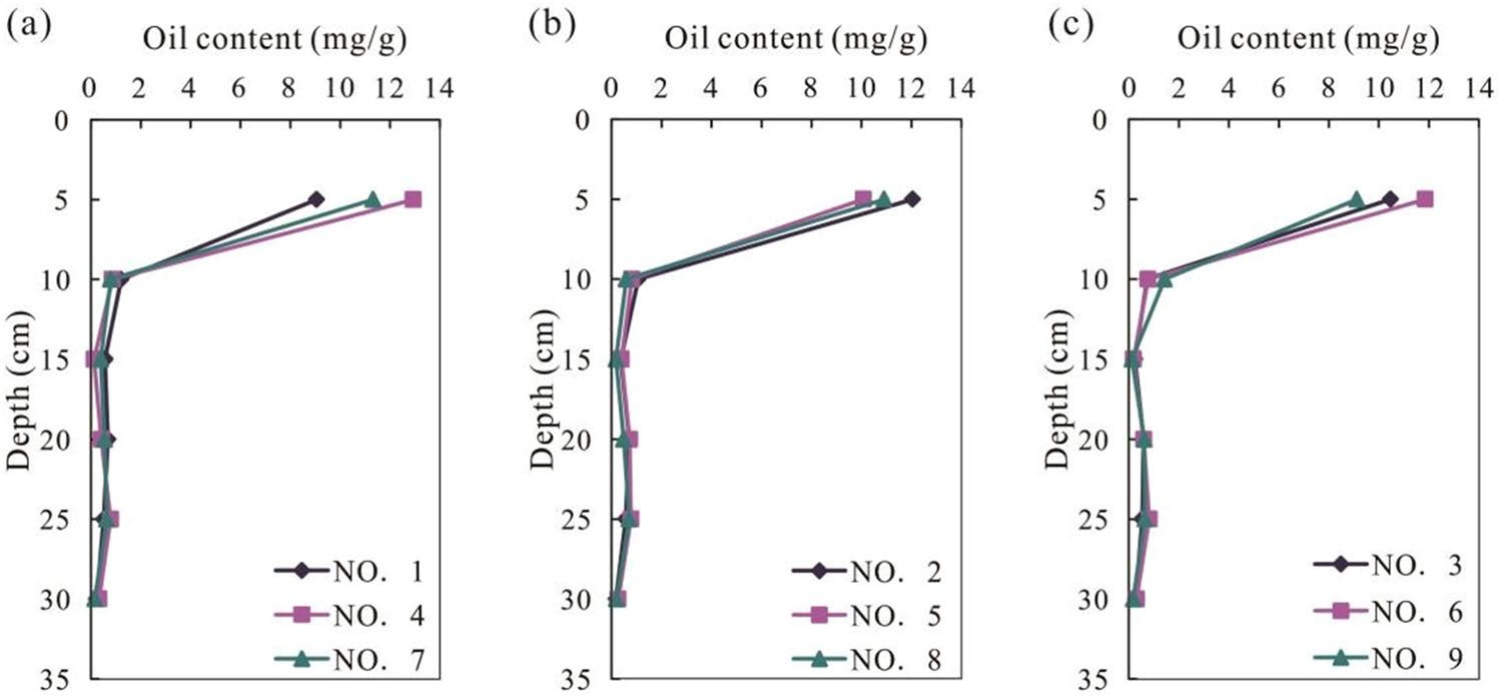
Oil content versus depth plot from disturbed soil column. (**a**) Oil content versus Depth plot for 1600 mL pouring amount; (**b**) Oil content versus depth plot for 4800 mL pouring amount; (**c**) Oil content versus Depth plot for 8000 mL pouring amount.

The experimental results are shown in Fig. 3 (decreased background value) and Table 3. At 5 cm depth, the oil content in the soil ranges from 9.06 to 12.94 mg/g. The oil content of all homogeneous soil column decreases rapidly between 5 and 10 cm. At 10 cm depth, the oil content in the soil decreases to 0.59–1.43 mg/g. The concentration of the pollutant is very small in the range of 15–25 cm, which is a trace, and the maximum content of the contaminants is 0.83 mg/g. The maximum migration depth of the oil is 25 cm.

**Table 3:** Oil content of the soil from different depth of disturbed soil.

Depth (cm)	1	2	3	4	5	6	7	8	9	Blank
5	9.06	12.05	10.46	12.94	10.07	11.86	11.32	10.91	9.12	8.074
10	1.21	1.07	0.74	0.86	0.81	0.78	0.79	0.59	1.43	0.991
15	0.56	0.37	0.24	0.13	0.40	0.19	0.40	0.18	0.13	0.107
20	0.68	0.71	0.60	0.42	0.73	0.60	0.55	0.49	0.62	0.397
25	0.51	0.57	0.54	0.79	0.78	0.82	0.64	0.71	0.65	0.558
30	0.23	0.19	0.27	0.32	0.26	0.31	0.16	0.20	0.18	0.1

The composition of pollutant from depths is shown in Table 4 and Continued Table 4 (The data of No. 5 for 15 cm, No. 2 for 25 cm in Table 4, and No. 7 for 25 cm in Continued Table 4 are invalid due to experimental reasons, so they are not put in the table). Pollutants from 5 cm depth, have more complex carbon components, containing C_10_–C_13_ components, mainly C_15_–C_19_, and lacking hydrocarbons greater than C_25_. Pollutants from 10 cm depth, lack of light components, are distributed in C_13_ and C_28_, mainly in C_15_–C_19_. Pollutants from 15 cm depth, also lack of light components, are distributed in C_16_–C_28_, mainly in C_16_–C_22_, with minor heavy carbon components. Pollutants from 20 cm depth contain C_17_ and C_21_, lacking light and recombination components. C_28_ was detected in other samples. Pollutants from 25 cm depth only contain C_17_–C_22_, lacking light and heavy components. The contents of pollutants from the same depth of soil columns are basically similar, in which C_17_ content of petroleum hydrocarbon components are the highest, followed by C_16_, C_18_ and C_19_. With the increase of soil depth, the light carbon components above C_17_ gradually disappear. The content of pollutants in different depths of soil was tested, and it was found that the content of C_17_ increased with the increase of depth, proved it has strong migration ability in soil column.

**Table 4:** Petro-hydrocarbon component of pollutant.

Component (%)	2	5	8	2	5	8	2	8	2	5	8	5	8	
	5 cm			10 cm			15 cm		20 cm			25 cm		
C10	17.44		3.62											
C11	23.43	1.76	7.38											
C12	15.33	4.28	10.98											
C13	7.51	5.60	13.79											
C14	1.86	7.24	14.92		4.09									
C15	4.67	10.79	0.66		9.57	4.47								
C16	6.79	6.46	13.37	9.63	1.35	9.49	60.31	7.48						
C17	5.24	13.24	1.37	16.70	15.14	13.64	23.21	15.97	100	20.39	27.39	34.38	20.70	
C18	2.29	10.53	0.71		1.21	15.36	5.25	1.87						
C19	2.45	9.15	9.42	22.26	14.99	13.93	1.23	22.00			4.51	11.77	16.91	
C20		5.75	7.16		11.79	10.83		0.87			17.44	7.76	8.80	
C21		5.24	6.35		12.45	1.67		15.79			50.66	46.10		
C22		2.19	4.38		7.47	8.38		1.12					53.6	
C23		1.33	0.32	2.51	1.84	0.54								
C24		2.26	2.78		7.21	0.58		6.18						
C25			2.01		1.04	5.18								
C26					0.34	2.86								
C27					0.81	2.16								
C28					0.65	0.94		1.63		29.4				

Component (%)	1	3	7	1	3	7	1	3	7	1	3	7	1	3
	5 cm			10 cm			15 cm			20 cm			25 cm	
C10														
C11	9.32													
C12	35.8	2.53	1.03											
C13	29.96	8.21	3.03											
C14	15.31	14.15	5.52											
C15		18.3	9.05	5.4										
C16		15.45	9.93	9.57		6.08	4.53	25.6						
C17	3.58	12.37	12.41	13.82	100	16.52	11.38	39.92	27.77	45.26	100	34.22	30.54	100
C18		5.4	11.11	15.05		2.95	17.64		10.02	15.47		2.74	25.2	
C19		3.05	10.68	15.32		16.55	18.22		1.47	14.62		11.07	28.32	
C20		1.79	4.51	1.46		2.32	16.59		14.95	1.88		23.98		
C21			4.15	11.19		1.07	14.25		8.68	9.13		28	15.93	
C22			3.23	6.73		13.36	10.4		7.92					
C23			5.43	7.71		1.39	1.07		5.33					
C24			0.28	0.7		1.63	0.44		5.51					
C25			3.44	0.34		10.81	0.33		2.33					
C26			1.67			8.32	0.37		3.5					
C27						2.22			1.83					
C28						9.44	2.76		3.69					

**Figure 4:**
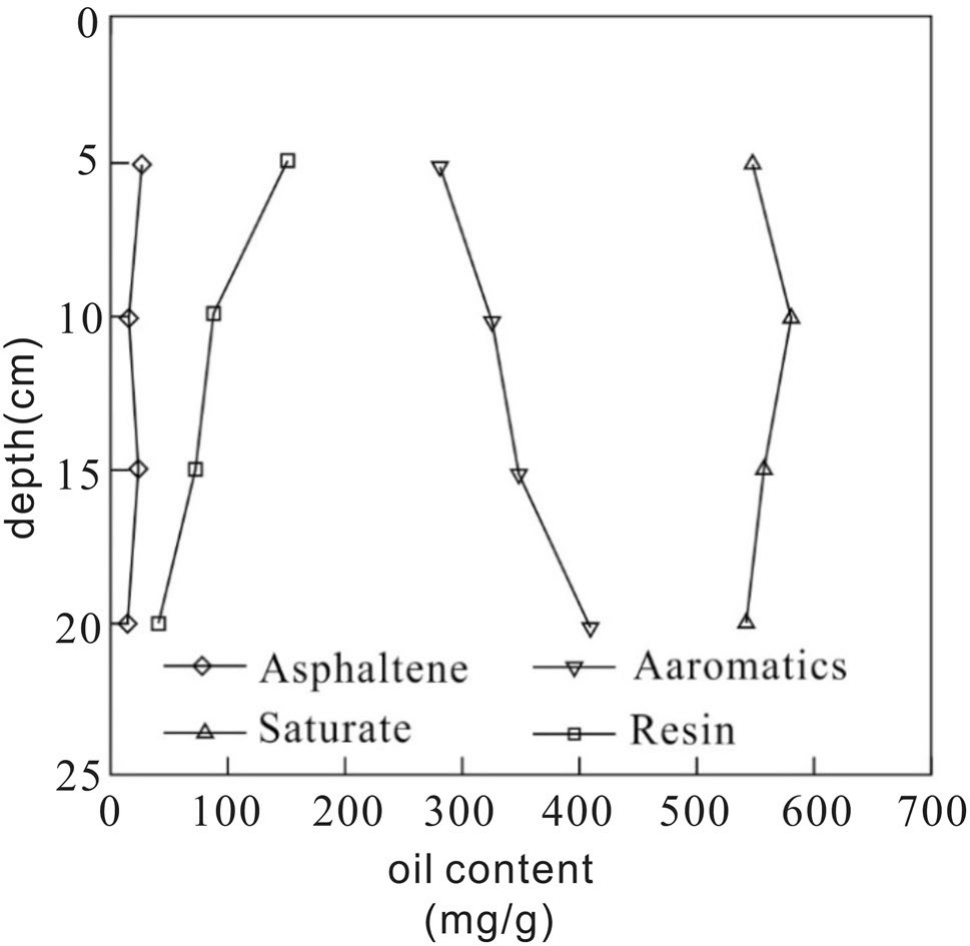
Four-component analyses of petroleum hydrocarbon components in the 7th soil column.

The migration rate of components of pollutants in soil is different, which needs to be separated. As shown in Fig. 4, taken NO. 7 soil column for an example, the four-component analysis of crude oil shows the component of pollutants from different depths vary obviously.

The components of crude oil are saturating of 547 mg/g, aromatics of 278 mg/g, resin of 151 mg/g, and asphaltene of 24 mg/g. With the increase of depth, the relative content of aromatics increases to 408 mg/g, and saturate and asphaltene change little, and the relative content of resin decreases to 39 mg/g.

### Results of undisturbed soil column experiment

**Figure 5:**
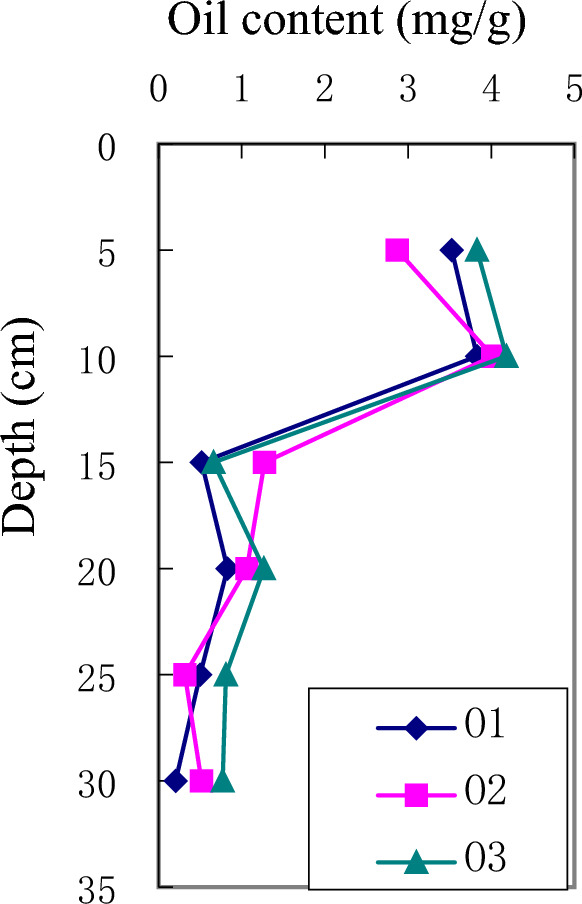
Oil content versus depth plot from undisturbed soil column.

Undisturbed soil experimental results were shown in Table 5 and Fig. 5 (decreased background value). Oil content patterns show certain differences between disturbed soil samples and undisturbed soil samples. The pollutant contents were mainly concentrated in the first 5 cm (2.87, 3.53, 3.83 mg/g respectively) and 10 cm (3.83, 4.02 4.18 respectively). The maximum detection depth of pollutants is 25 cm.

**Table 5:** Oil content of the soil from different depth of undisturbed soil.

Depth (cm)	O1	O2	O3
5	3.53	2.87	3.83
10	3.83	4.02	4.18
15	0.52	1.27	0.66
20	0.83	1.06	1.27
25	0.50	0.32	0.81
30	0.21	0.52	0.77

**Figure 6:**
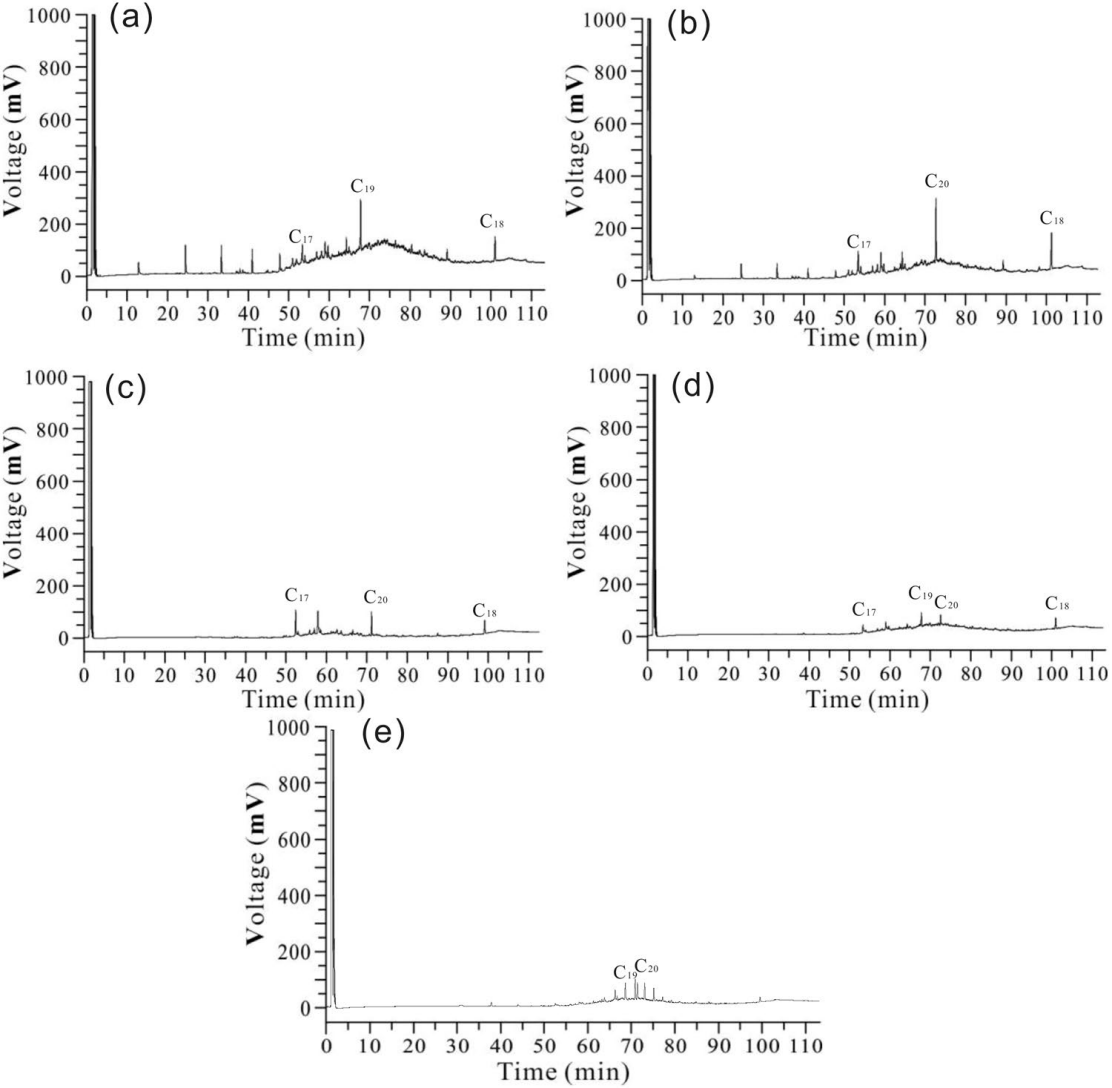
Gas chromatogram of oil pollutants in disturbed soil. (**a**) Gas chromatogram at 5 cm; (**b**) Gas chromatogram at 10 cm; (**c**) Gas chromatogram at 15 cm; (**d**) Gas chromatogram at 20 cm.

The results of pollutant component from the NO. 2 soil column are show in Table 6 and Figure 6. Pollutants from 5 cm depth, composed of C_12_–C_19_ components. Pollutants from 10 cm depth, are distributed in C_13_ and C_22_. Pollutants from 15, 20 and 25 cm depths, lack of light components, are mainly distributed in C_17_–C_20_.

**Table 6:** Oil content of the soil from different depth of undisturbed soil sample NO. O2.

Component (%)	5 cm	10 cm	15 cm	20 cm	25 cm
C12	4.10				
C13	8.64	6.77			
C14	11.65	7.27			
C15	10.27	4.88			
C16	7.70	3.39			
C17	8.03	18.80	37.70	17.74	12.45
C18	5.40	4.05	29.82	18.45	49.28
C19	36.69	8.35		38.82	13.45
C20		38.98	32.47	24.99	24.82
C22		5.39

The experimental results show that the pollutants in shallow soil are mainly concentrated between C12 and C22, and the relative contents of C19 and C20 are relatively high. With the increase of the depth of the soil column, the components of the pollutants gradually decrease, the low-carbon components disappear, and the pollutants are in the middle group.

**Figure 7:**
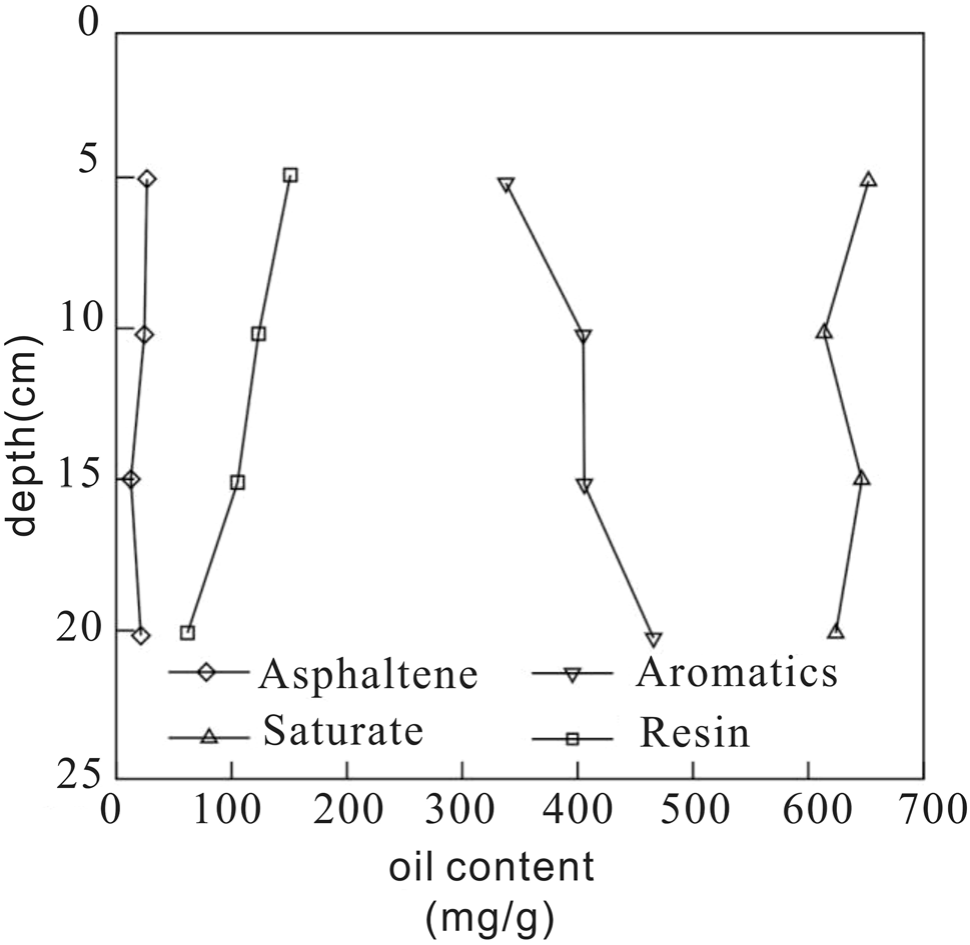
Four-component analyses of petroleum hydrocarbon components in the O_2_ soil column.

As shown in Fig. 7, taken NO. O2 soil column for an example, the four-component analysis of crude oil shows similar pattern with that of disturbed soil. With the increase of depth, the relative content of aromatics increases. The saturate and asphaltene don’t show obvious change, and the relative content of resin decreases.

## Discussion

### Comparison of experimental results between disturbed soil and undisturbed soil

The migration ability of pollutants in soil is weak, pollutants fall to the ground, and the surface soil is polluted first. Compared with disturbed soil, the structure of undisturbed soil is complex. During the migration of petroleum pollutants in the soil column, influenced by precipitation and soil properties, the concentration of pollutants changes negatively exponential with the increase of depth. Compared with the disturbed soil column, the migration law of the shallow layer of the undisturbed soil column changes due to the different pore distribution and water content. With the increase of leaching times, the oil content curve no longer has an obvious linear relationship, and within the same time after pollution, the migration rate of pollutants in the undisturbed soil is slow, while the larger porosity of the disturbed soil sample leads to a relatively fast migration rate. In addition, as the depth of the disturbed soil column increases, the light components gradually decrease, and the relative content of the heavy components does not change much. As the depth of the undisturbed soil column increases, the light components gradually decrease, and the heavy components larger than C22 do not migrate to the depth of the soil column.

### Effects of leached water amount and oil concentration on pollutant concentrations

From Fig. 3, it can be observed that leached water amount and pollutant content do not show significant relationship. In soil, crude oil migration is affected by diffusion, adsorption–desorption and physicochemical interaction between oil and water. There are few water-soluble components in petroleum. If the soil capillary was full of water, it will hinder the migration of pollutants^15^. Moreover, the bound water around soil particles will prevent pollutant components contacting with the particles, and reduce the adsorption of pollutants in the soil^15^. However, if the capillary is filled with water and oil, the oil and water will move towards the lower pressure end of the capillary. According to our experimental results, leached water amount has little influence on oil migration, because of the immiscibility of oil and water, only a very small amount of oil flowing in water will dissolve, so the oil is mainly adsorbed in the soil. That is to say, most oil are exist in the form of adsorption in soil.

Theoretically, the concentration of pollutants has a certain influence on the dispersion degree of soil particles. In this contribution, pollutant concentration is not affected by crude oil concentration, indicating pollutants do not migrate by the mechanical diffusion driven by concentration differences or gravity. We add 15 g crude oil at most, and its volume is 17.44 cm^3^ according to the density of 0.86 g/mL. The inner diameter of the soil column is 8.4 cm, and the minimum porosity of the soil is 38.44%. For the soil column with a height of 5 cm, the pore volume will be 106.46 cm^3^, which is far larger than the volume of oil, resulted an unsaturation. As argued by Zhao et al.^16^, under our experimental conditions, i.e. oil unsaturation, neither leached water amount nor pollutant concentration is the main factor controlling the oil migration pattern.

### Effects of porosity on pollutant concentrations

After pouring, the oil content at 10 cm has decreased to about 1 mg/g and 4 mg/g for disturbed soil-column and undisturbed soil-column. Affected by porosity and soil homogeneity, in the soil column with a depth of more than 5 cm, the pollutant contents from undisturbed soil-column were slightly higher than that of disturbed soil-column. As a porous medium, soil is a multiphase system containing solid and liquid phases, and has a large area ratio. The petroleum pollutants exist mainly in fine particles in the water. Therefore, the adsorption of soil to pollutants is mainly due to the adhesion force on the surface of soil particles, as well as part of Van der Waals force and electrostatic attraction^17^. The intensity of adsorption is mainly determined by the surface area of soil particles and the polarity of oil components, and the greater the polarity means the easier the adsorption^8^. Because the undisturbed soil is directly filled into the soil column according to the sampling depth after sampling, and the physical properties are not affected and not disturbed. After the homogeneous soil is sampled and ground, the soil sample becomes homogeneous, the physical properties are affected, the soil bulk density becomes larger, and the entry of petroleum pollutants is affected by the porosity, permeability and absorbability of the soil. The macromolecular components of petroleum pollutants enter the soil and adsorb on the upper soil surface, blocking soil pores. Soil particles and oil are always a continuous dynamic adsorption–desorption process, in which the gradual decrease of adsorption capacity leads to the gradual separation of components. Finally, colloids and asphaltenes exhibit resistance to degradation and will adhere to soil cracks, thereby adsorbing on the soil surface. Therefore, the inflow of aromatic hydrocarbons and alkane compounds with relatively strong desorption capacity is relatively increased, which will penetrate into deeper soil.

### Effect of different crude oil components on migration pattern

Different components of petroleum pollutants have different physical and chemical properties and different migration ability in soil. As for aromatic hydrocarbons, those with low molecular weight can be dissolved in water, while those with high molecular weight are not easily dissolved in water^18,19^, so naphthalene with lower molecular weight is soluble in water, anthracene and phenanthrene have moderate solubility, and components with molecular weight greater than 228 are hardly soluble in water. The water solubility of saturates is less than that of aromatic hydrocarbons, in which saturates above C_8_ are almost insoluble in water and have the weakest migration ability. Therefore, aromatic hydrocarbons are the most soluble among the four components of crude oil, and their migration ability is greater than other components in soil. With the increase of soil depth, the relative content of aromatic hydrocarbons increases, and the content of aromatic hydrocarbon is high, the migration ability is strong, and it may also be affected by biodegradation, which affects the migration of hydrocarbon substances. The migration ability of low carbon component is weaker than the medium carbon component. The components with high carbon number can neither disappear through volatilization nor migrate to deeper underground, so the pollutant content in middle-layer soil column is significantly higher than that in deep soil column. The relative contents of each component from high to low are saturates, aromatic hydrocarbons, resin and asphaltene in the soil.

## Conclusions

In this contribution, based on soil column experimental experiment, the migration pattern of petroleum pollutants in soil is studied, and the conclusions are listed as follow:

1. The pollution time has a certain influence on the vertical migration depth of pollutants. The pollution time is long, the migration depth of pollutants can reach 25 cm, and the pollution time is short, and the migration depth is about 15 cm. With the increase of pollution time, the oil content at 5 cm of soil column increases, and the oil content below 10 cm decreases obviously.
2. By comparing the migration of pollutants under different leaching rates, it can be seen that water content has a certain influence on the migration of pollutants. When the water content in soil increases, water molecules will adhere to the soil surface, which will cause competitive adsorption of pollutants, and the migration speed of oil in soil will be faster, which will further reduce the oil content in the upper soil. When the soil water content is low, the migration speed of oil will slow down, because the lack of water will limit the migration of oil.
3. Under the unsaturation condition, the pollution concentration doesn’t show obvious influence on the migration depth of pollutants. Porosity also has a certain influence on the migration of pollutants.
4. The relative contents of each component from high to low are saturates, aromatic hydrocarbons, resin and asphaltene in the soil. With the increase of soil column depth, the relative content of aromatic hydrocarbons increases, while the relative content of resin decreases, while the saturates and asphaltene change little.
5. Through the research of this paper, the migration law of oil pollution in soil is mastered, which provides theoretical guidance for the treatment of oil field soil pollution. According to the experimental data of soil migration law, a mathematical model can be established to provide guidance for the treatment of soil oil pollution.

### Supplementary Information

Supplementary Information.

## Data Availability

All data generated or analyzed during this study are included in this published article [and its supplementary information files].
